# Editorial Considerations on the New Clinical and Surgical Perspectives of Brazilian Cardiology

**DOI:** 10.5935/abc.20150094

**Published:** 2015-08

**Authors:** Paulo Roberto B. Evora, Alfredo J. Rodrigues

**Affiliations:** Departamento de Cirurgia e Anatomia da Faculdade de Medicina de Ribeirão Preto, Ribeirão Preto, SP - Brazil

**Keywords:** Cardiology/trends, Case-Control Studies, Cardiovascular Surgical Procedures/trends, Evidence Based Practice/trends

The *Arquivos Brasileiros de Cardiologia* (ABC) has been the official publication of the Brazilian Society of Cardiology for more than 60 years, and has proved to be one of the most prestigious scientific journals in Latin America, approaching all aspects of cardiology, including the surgical ones. In August 1986, the *Revista Brasileira de Cirurgia Cardiovascular* (RBCCV), specifically directed to the surgical aspects of heart diseases, began to be published. Thus, the editorial policy of the ABC has progressively privileged publications focused on the clinical treatment of cardiovascular diseases over surgical publications of common interest to cardiologists and cardiac surgeons^1^. Despite the substantial changes in the treatment of heart diseases, deriving from the appearance of new drugs and percutaneous devices, cardiac surgeries are still the treatment of choice to a substantial number of heart diseases, mainly the congenital, valvular and even coronary ones, whose onus of surgery indication is invariably on cardiologists. Thus, those specialists should be aware of several aspects of cardiac surgery, mainly those concerning their risks and results, so that they can base their indications on evidence.

The great majority of cardiologists might know that the use of bilateral internal thoracic arteries (BITA) for coronary artery bypass grafting improves survival as compared to the use of only one single internal thoracic artery (ITA)^2^. Thus, the optimization of arterial grafts has been widespread with the use of the radial artery (RA) and composite grafts. However, only few professionals are aware of the controversies involving risky situations, possible limitations to the use of BITA^3^, the patency index of RA grafts and their influencing factors. The RA was the second arterial graft introduced in the clinical practice for coronary artery bypass grafting, and has drawn the interest of Brazilian surgeons since the 1990s^4^. Skeletonization of the left ITA (LITA) can favorably change the flow capacity of the graft, leading to the assumption that the behavior of the RA, as a coronary graft, is similar to that of the skeletonized LITA. Considering the potential role of the RA as a second option of coronary grafting and the concept of complete coronary artery bypass grafting with the exclusive use of arterial grafts, Bonini et al^5^ have carried out a prospective randomized study comparing 40 patients distributed into two groups. In group I, skeletonized RA was used in 20 patients, and, in group II, RA with adjacent tissues was used in 20 patients. After the surgical procedure, flow velocity was measured. The results showed that the morphological and pathological characteristics, as well as the hemodynamic performance of the free RA grafts, regardless of being skeletonized or having adjacent tissues, were similar. However, a larger number of non-obstructive lesions was observed in the group with the RA graft preserving the adjacent tissues^5^.

In addition, small is the number of cardiologists aware of the models to calculate the perioperative risk for morbidity and mortality available and popular among cardiac surgeons^6^, as well as of their limitations, mainly regarding the most recent options of percutaneous therapies^6,7^. The applicability of risk scores to cardiac surgery is another relevant international matter, but not well defined in centers outside North America and Europe. Garoffalo et al^8^ have assessed the ability of the Parsonnet-Bernstein 2000 score and EuroSCORE to predict the in-hospital mortality of patients undergoing cardiac surgery at a reference hospital in Brazil, and have identified risk predictors. The use of those scores has underestimated in-hospital mortality, suggesting inadequate preoperative assessment of the patients undergoing cardiac surgery. That study has stressed the need to develop local scores based on the reality of the populations to better assess the risk of cardiac surgery^3^.

Considering the controversies about the ideal method of coronary artery bypass grafting for patients on dialysis, Herzog et al^9^, in the United States, have compared long-term survival of patients on dialysis after angioplasty, coronary stenting and coronary artery bypass grafting. That retrospective study has reported better long-term survival of patients on dialysis after coronary artery bypass grafting than after percutaneous coronary intervention and coronary stenting, emphasizing the relatively poorer results of patients with diabetes. That study has supported the need to develop large clinical registries and prospective studies on coronary revascularization procedures for patients on dialysis^9^. Miranda et al^10^ have approached that important question, analyzing retrospectively 50 consecutive and non-selected patients on dialysis, undergoing coronary artery bypass grafting at a tertiary university-affiliated hospital between 2007 and 2012. Those authors have shown that coronary artery bypass grafting is feasible in patients on dialysis, although followed by high morbidity and in-hospital mortality. In addition, they have emphasized the frequent exclusion of that group of patients from large cardiac studies. That detail might even contribute to the difficulty in selecting a better approach and to the still modest surgical results as compared to those of patients with preserved kidney function^10^.

It is therefore evident that, although the ABC prioritizes articles on clinical therapies and their guidelines, the restriction upon articles with a surgical bias can deprive cardiologists from essential information to support their clinical decision-making, especially regarding the case-to-case analysis in daily practice. It is worth noting the publication in the ABC of several articles of common interest to both cardiac surgeons and cardiologists in recent years, therefore providing a relevant service to cardiology and cardiovascular surgery.
